# Retrospective Cohort Study of Caveolin-1 Expression as Prognostic Factor in Unresectable Locally Advanced or Metastatic Pancreatic Cancer Patients

**DOI:** 10.3390/curroncol28050303

**Published:** 2021-09-09

**Authors:** Alessandro Bittoni, Riccardo Giampieri, Federica Pecci, Giada Pinterpe, Alessandra Mandolesi, Michela Del Prete, Antonio Zizzi, Sonia Crocetti, Carolina Liguori, Giulia Mentrasti, Luca Cantini, Chiara Pellei, Renato Bisonni, Marina Scarpelli, Rossana Berardi

**Affiliations:** 1 Medical Oncology Unit, Azienda Ospedaliero-Universitaria Ospedali Riuniti Umberto I-GM Lancisi-G Salesi, Università Politecnica delle Marche, 60126 Ancona, Italy; alessandro.bittoni@ospedaliriuniti.marche.it (A.B.); riccardo.giampieri@ospedaliriuniti.marche.it (R.G.); s1086870@pm.univpm.it (F.P.); s1092486@pm.univpm.it (G.P.); s1087805@pm.univpm.it (S.C.); s1102602@pm.univpm.it (C.L.); s1090046@studenti.univpm.it (G.M.); luca.cantini@pm.univpm.it (L.C.); chiarapellei@libero.it (C.P.); 2 Department of Pathological Anatomy and Histopathology, Azienda Ospedaliero-Universitaria Ospedali Riuniti Umberto I-GM Lancisi-G Salesi, 60126 Ancona, Italy; alessandra.mandolesi@ospedaliriuniti.marche.it (A.M.); antonio.zizzi@asl.brindisi.it (A.Z.); marina.scarpelli@ospedaliriuniti.marche.it (M.S.); 3 Medical Oncology Unit, Ospedale A. Murri, 63900 Fermo, Italy; micheladelprete@gmail.com (M.D.P.); renato.bisonni@sanita.marche.it (R.B.); 4 Medical Oncology Unit, Ospedale Madonna del Soccorso, 63074 San Benedetto del Tronto, Italy

**Keywords:** Caveolin-1, pancreatic cancer, 1st line chemotherapy, prognosis

## Abstract

Caveolin-1 (Cav-1) plays a key role in various neoplastic diseases and is upregulated in different cancers, including pancreatic ductal adenocarcinoma (PDAC). Furthermore, Cav-1 is critical for the uptake of albumin as well as nab-paclitaxel in PDAC cells. Here, we investigated the prognostic impact of Cav-1 expression in a cohort of 39 metastatic PDAC patients treated with different first-line chemotherapy regimens. We also assessed the predictive value of Cav-1 in patients treated with gemcitabine and nab-paclitaxel. Cav-1 expression was evaluated by immunohistochemistry staining in neoplastic and stromal cells, using metastatic sites or primary tumor tissue specimens. Higher levels of Cav-1 expression were associated with significantly worse overall survival (OS) and progression-free survival (PFS). No differences in OS were found between patients treated with gemcitabine + nab-paclitaxel vs. other chemotherapy options. Multivariate analysis for OS and PFS confirmed the independent prognostic role of Cav-1 expression. Our study evidenced a negative prognostic role of Cav-1 in patients affected by metastatic/locally advanced unresectable PDAC. Moreover, Cav-1 expression seems not to predict different response rates to different types of first-line treatment. Future prospective trials will be necessary to confirm the prognostic role of Cav-1 and explore Cav-1 specific inhibitors as a therapeutic option for advanced PDAC patients.

## 1. Introduction

Despite improvements in surgical and medical treatments, pancreatic ductal adenocarcinoma (PDAC) still represents one of the deadliest malignancies. With a five-year overall survival (OS) still under 10% and an increasing incidence over the last decade, PDAC is projected to be the second leading cause of cancer-related death by 2030 [1].

Late diagnosis and rapid progression of the disease together with resistance to chemotherapy and radiotherapy contribute to PDAC dismal prognosis. Moreover, PDAC is characterized by a dense fibrotic stroma, which represents a physical barrier for therapies and also has an established role in promoting cancer progression. Gemcitabine has been the mainstay of treatment of both resected and metastatic PDAC for many years while more recently, the combination regimens of either 5-fluorouracil (5-FU)/leucovorin with irinotecan and oxaliplatin (scheme FOLFIRINOX), or gemcitabine and nanoparticle albumin-bound paclitaxel (nab-paclitaxel) have become the standard of care in different settings on the basis of phase III clinical trials showing superior outcomes with either of the combinations over gemcitabine alone [2,3,4]. This scenario highlights the need for identification of biomarkers associated with disease prognosis with utility to select patients for different treatments.

Caveolin-1 (Cav-1) is the principal structural component of caveolae (flask-shaped invaginations of plasma membrane) and plays a key role in cellular endocytosis, lipid homeostasis, and signal transduction, serving both to compartmentalize and regulate cellular signaling [5]. Caveolae and caveolins had a crucial role in various human pathobiological conditions, in particular cardiovascular and neoplastic disease, as reported by Zhang et al. in Caveolin-deficient mouse models [6]. Cav-1 is upregulated in different cancers, including PDAC, lung cancer, and breast cancer, and several studies have demonstrated an association between Cav-1 expression and invasion, distant metastasis, and poor prognosis [7,8,9].

Furthermore, Cav-1 expression has been correlated with resistance to radiotherapy and chemotherapy in PDAC cells [10,11]. In particular, tumor Cav-1 knock-down significantly reduced beta1 integrin expression and Akt phosphorylation, induced Caspase 3- and Caspase 8-dependent apoptosis, and enhanced the radiosensitivity of 3D human pancreatic cell cultures [12].

Interestingly, recent studies have shown that Cav-1 may play a dual role in PDAC biology depending on the site of expression of the protein, tumor type, and stage of the disease. In particular, while its expression in cancer cells has been associated with an aggressive phenotype and poor prognosis, the loss of Cav-1 in the tumor stroma has been associated with chemoresistance and increased tumor growth [13].

Pre-clinical studies have shown that Cav-1 is critical for the uptake of albumin as well as nab-paclitaxel in PDAC cells, causing a subsequent apoptotic response in tumor cells in vitro. Moreover, Cav-1 expression correlates positively with sensitivity to this drug, representing a promising predictive biomarker for nab-paclitaxel [14].

In this study, we investigated the prognostic impact of Cav-1 expression in a cohort of locally advanced or metastatic PDAC patients treated with different first-line chemotherapy regimens, including gemcitabine plus nab-paclitaxel, FOLFIRINOX scheme, and gemcitabine monotherapy. We also assessed the predictive value of Cav-1 in patients treated with gemcitabine and nab-paclitaxel.

## 2. Materials and Methods

### 2.1. Patients and Tissue Samples

A total of thirty-nine locally advanced or metastatic PDAC patients treated with first-line chemotherapy at the Oncology Departments of AOU Ospedali Riuniti “Umberto I–G.M. Lancisi–G. Salesi” (Ancona, Italy) and Ospedale “Augusto Murri” (Fermo, Italy) between April 2013 and January 2021 were included in this analysis. For each patient enrolled, we collected data concerning age, gender, ECOG PS (Performance Status) at the beginning of treatment, grading, TNM stage (8th edition), site of metastasis, and response to treatment (assessed by RECIST 1.1).

Data were retrospectively collected from medical chart reviews and electronic records. Patients consented to treatment and procedures. The study was conducted according to the guidelines of the Declaration of Helsinki and approved by the Institutional Ethics Committee of Azienda Ospedaliero Universitaria Ospedali Riuniti Umberto I-G.M. Lancisi-G. Salesi di Ancona (protocol code 214341).

### 2.2. Immunohistochemistry

**Figure 1:**
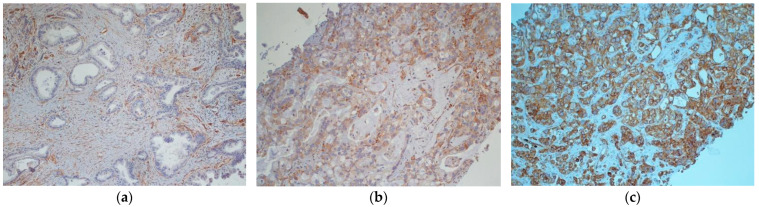
Examples of tumoral Caveolin-1 expression (**a**) Caveolin-1 negative sample (0) (**b**) Caveolin-1 positive sample (2+) (**c**) Caveolin-1 positive sample (3+).

Immunostainings were performed on conventional 5-μm-thick paraffin tissue sections on positively charged slides. After heat drying, to dewax and better unmask antigenic sites, an antigen retrieval solution was applied, incubating sections for 20 min in a Dako PT-Link autostainer (DakoCytomation, Glostrup, Denmark) with a Dako Target Retrieval solution (DakoCytomation), pH 9.0, at 750 KW for 20 min before antibody staining. Endogenous peroxidase activity was quenched, incubating the sections in 3% (*v/v*) hydrogen peroxide for 7 min at room temperature. Tissue sections were then incubated in a Dako Autostainer Link 48 with the Caveolin-1 rabbit polyclonal antibody (cat. N. 16447-1-AP, dil. 1:200, Proteintech, Rosemont, IL, USA). The antigen–antibody complex was subsequently visualized using the Envision⁄HRP Detection System kit peroxidase⁄DAB (DakoCytomation). Sections were counterstained with Mayer’s Hematoxylin (Bio-Optica SPA, Milano, Italy) and coverslipped with Paramount. Negative control slides omitting the primary antibody were included in the assay. We used tissue sections from a human lung cancer tissue as positive control. Capillary endothelial cells were used as internal positive controls in all tissue specimens examined. All evaluations were independently performed by two investigators who were blinded to the patient group. Immunostained cells for Cav-1 were enumerated in 10 representative and consecutive microscopic high-power fields (40X-HPF). Images were captured with a digital camera connected to a light mycroscope and a computer. We evaluated positivity immunoreactivity of Cav-1 in neoplastic and stromal cells. Fine needle aspiration biopsies (FNAB) samples were considered adequate for Cav-1 immunohistochemical analysis if contained a minimum of 100 viable tumor cells. Positivity immunostaining of Cav-1 is defined if the cells showed granular staining at the cell membrane and in the cytoplasm. The percentage of positive neoplastic cells was assessed, and the intensity of the immunostaining was scored as negative (0) (Figure 1a), weakly (1+), moderately (2+) (Figure 1b), or strongly (3+) (Figure 1c). Inconsistencies were discussed until an agreement was reached.

### 2.3. Statistical Analysis

Progression-free survival (PFS) was calculated by Kaplan–Meier method starting from the first day of chemotherapy until the first radiological/clinical sign of disease relapse, death or last follow-up visit for patients lost at follow-up. Overall survival (OS) was calculated by Kaplan–Meier method starting from the beginning of chemotherapy until date of death or last follow-up visit for patients lost at follow-up.

A log-rank test was used to assess differences among the strata whereas multivariate analysis was performed by Cox-proportional hazard regression. Association between categorical variables was assessed by Chi-square test. All analyses were performed with a level of statistical significance p set at 0.05. Statistical analysis was conducted using MedCalc software version 19.1 for Windows (Ostend, Belgium).

## 3. Results

### Patients’ Characteristics

Thirty-nine patients were eligible for analysis. Median age was 68 years old (range 31–86 years). Seventeen patients (43%) presented with locally advanced disease while 22 (67%) had metastatic disease. The majority of patients (76%) had liver metastases, 33% lymph node involvement, and 20% lung metastases. As first line treatment, 27 patients (69%) received gemcitabine plus nab-paclitaxel, five patients (12%) the combination regimens of 5-fluorouracil/leucovorin with irinotecan and oxaliplatin (FOLFIRINOX) or the combination regimens of 5-fluorouracil/leucovorin with oxaliplatin (FOLFOX), and seven patients (17%) were treated with gemcitabine as monotherapy. Patients’ clinical characteristics are summarized in Table 1.

Median OS of the whole cohort of patients was 11.21 months (95%CI:7.77–16.07) while median PFS was 5.02 months (95%CI:3.61–8.59). Evaluating the disease control rate (DCR) according to RECIST 1.1 criteria, 10 patients (25%) obtained disease stability (SD), eight patients (20%) partial response (PR), and 17 patients (43%) had disease progression (Table 2). Only nine of the 39 patients are still alive at the time of the present analysis and only five of 39 have not yet progressed under first-line treatment.

Cav-1 expression in the tumor samples was as follows: 13 in 39 patients were caveolin expression negative (0), two out of 39 patients were caveolin expression positive 1+, 16 in 39 patients were caveolin expression positive 2+, and the remaining seven patients had 3+ positive caveolin expression. One FNAB sample was excluded from the analysis due to the low cellularity (less than 100 viable tumor cells).

In 11out of 39 patients Cav-1 expression was assessed in biopsies taken from metastatic sites, whereas in 28/39 patients caveolin expression was assessed in biopsies taken from the primary tumor (Table 3).

**Figure 2:**
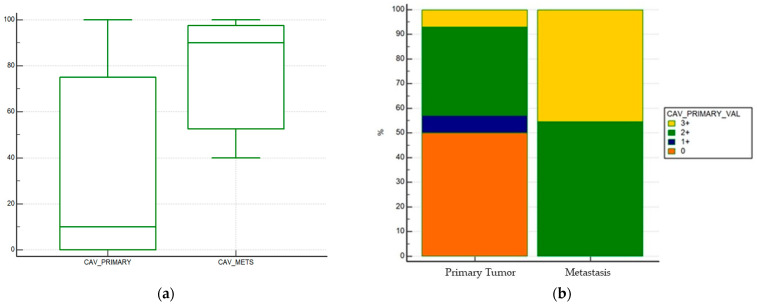
Differences in Caveolin-1 expression between primary tumor and metastases. (**a**) Differences in Caveolin-1 expression as a continuous variable. Median % of expression of primary tumor 10% vs. 90% of metastatic samples, *p* = 0.001. (**b**) Differences in Caveolin-1 expression by IHC score (0 vs. 1+ vs. 2+ vs. 3+, *p* = 0.0038).

Cav-1 expression was higher in metastatic samples rather than in histological samples taken from the primary tumor: percentage of tumor cells that were caveolin positive was higher in metastatic samples compared to primary tumor samples (median % of expression of primary tumor 10% vs. 90% of metastatic samples, *p* = 0.001) (Figure 2a).

When stratifying positivity in a semiquantitative fashion (0 vs. 1+ vs. 2+ vs. 3+ expression), a statistically significant difference in positivity was found (*p* = 0.0038) (Figure 2b).

**Figure 3:**
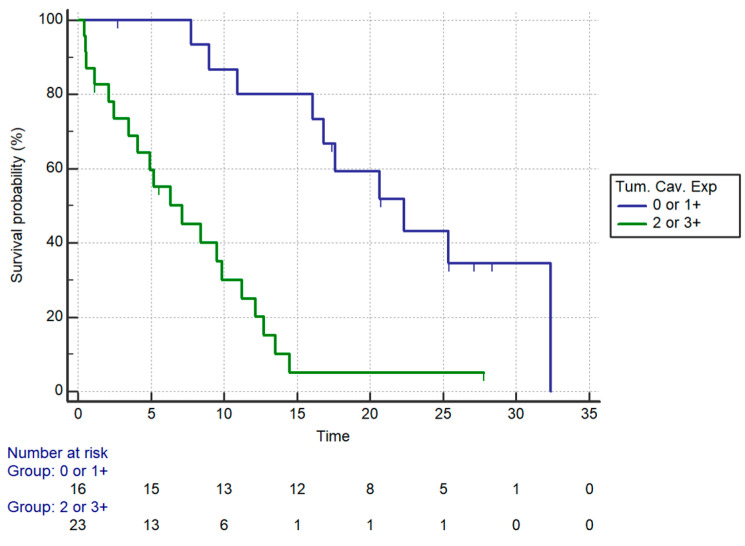
OS by positive (0 and 1+ vs. 2+ and 3+) Caveolin-1 expression in the whole cohort. Median OS: 7.15 vs. 22.30 months (HR: 5.35, 95%CI: 2.38–12.05, *p* = 0.0001).

OS was significantly different in patients stratified by intratumoral caveolin expression (*p* = 0.0001). Stratifying patients by caveolin expression (2+ and 3+ vs. 0 and 1+), a statistically significant impact on overall survival was seen (median OS: 7.15 vs. 22.30 months respectively for higher vs. lower levels of caveolin expression, HR: 5.35, 95%CI: 2.38–12.05, *p* = 0.0001) (Figure 3, Table 4).

When assessing differences in OS in patients treated with different first-line chemotherapy regimens, we failed to demonstrate a different prognostic role of Cav-1 expression when comparing patients treated with gemcitabine + nab-paclitaxel vs. those treated with other chemotherapy options. In particular, in patients treated with nab-paclitaxel plus gemcitabine (27 in 39 patients) Cav-1 expression was associated with significantly worse OS (HR = 5.01, 95%CI: 1.85–13.52, *p* = 0.0015). In patients treated with other regimens (12 in 39 patients), a similar negative prognostic effect was seen (HR = 5.59; 95%CI 1.35–23.05 *p* = 0.017) (Table 2).

**Figure 4:**
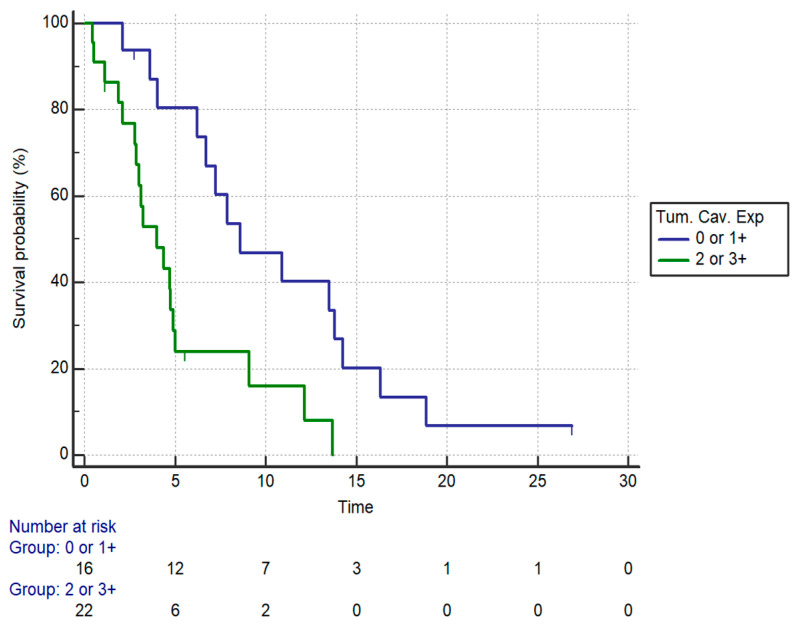
PFS by positive (0 and 1+ vs. 2+ and 3+) Caveolin-1 expression in the whole cohort. median PFS: 4.0 vs. 8.59 months (HR:3.12, 95%CI:1.44–6.72, *p* = 0.0037).

PFS was also significantly different in patients stratified by intratumoral caveolin expression (*p* = 0.0037). Stratifying patients by Cav-1 expression (2+ and 3+ vs. 0 and 1+), a statistically significant impact on PFS was seen (median PFS: 4.0 vs. 8.59 months, HR:3.12, 95%CI:1.44–6.72, *p* = 0.0037) (Figure 4, Table 5). When we analyzed separately the outcomes of patients with locally advanced PDAC and with metastatic disease, the prognostic role of tumoral Cav-1 was confirmed in both settings. In particular, high Cav-1 expression was associated with significantly worse OS both in patients with locally advanced disease (median OS: 7.15 vs. 16.82 months, HR:8.26, 95% CI: 2.16–31.45, *p* = 0.002) and in metastatic patients (median OS: 5.18 vs. 22.33 months, HR:4.24, 95%CI:1.45–12.33, *p* = 0.008). PFS was also significantly shorter in locally advanced patients with high Cav-1 expression compared to patients with low expression (4.72 vs. 7.87 months, HR:3.85, 95%CI:1.18–12.54, *p* = 0.002).

Patients with higher levels of caveolin expression did not have significantly higher rates of liver metastases compared to those with lower levels (*p* = 0.23). There were also no differences in terms of higher rates of peritoneal metastases (*p* = 1), bone metastases (*p* = 0.50), or lung metastases (*p* = 1).

Patients with higher levels of expression of Cav-1 had also worse ECOG PS (*p* = 0.01). In particular, 12 in 16 patients with caveolin expression 0 or 1+ had ECOG PS: 0 compared to only six in 23 patients with Cav-1 expression 2 or 3+. On the other hand, only one patient with caveolin expression 0 or 1+ had ECOG PS:2 compared to six in 23 patients with Cav-1 expression 2 or 3+.

When assessing the impact of other stratifying factors, only first-line ECOG PS (*p* = 0.0012) was associated with a statistically significant impact on OS (Table 2). For PFS, type of treatment received (polychemotherapy with gemcitabine plus nab-paclitaxel or FOLFIRINOX scheme vs. monotherapy with gemcitabine) (*p* < 0.0001) and ECOG PS (*p* = 0.0004) were confirmed to be associated with different PFS (Table 3).

Multivariate analysis for OS confirmed that Cav-1 expression was the only factor that maintained its independent role as prognostic factor (HR:4.96, 95%CI:2.02–12.06, *p* = 0.005) (Table 2).

Multivariate analysis for PFS confirmed that Cav-1 expression (HR:2.81, 95%CI:1.17–6.76, *p* = 0.019) and type of treatment (polychemotherapy vs. monotherapy, HR:0.30, 95%CI:0.10–0.90, *p* = 0.0342) were the only factors that maintained their independent role on PFS (Table 5).

We also assessed stromal Cav-1 expression in tumor samples from core biopsies and surgical tumor samples. Stromal Cav-1 expression was not associated with clinical outcomes of patients. In particular, comparing patients with high (2+ or 3+) vs. low (0 or 1+) stromal Cav-1 expression, we found no significant difference in terms of PFS (HR:1.51, 95%CI:0.25–3.97, *p* = 0.39) or OS (HR:2.15, 95%CI:0.75–6.13, *p* = 0.47).

## 4. Discussion

PDAC is one of the leading causes of cancer mortality in developed countries and one of the most lethal tumors worldwide in both sexes, with a higher prevalence in men. The incidence and mortality rates are correlated with increasing age and almost 90% of all deaths are registered in those over 55 years old [15].

Despite the growing knowledge about its biology, therapeutic management of PDAC patients achieved relatively modest results.

Partial explanation of this failure has to be traced back to the lack of other treatment options besides chemotherapy. Only a small proportion of patients who detain the germline breast-cancer susceptibility gene 2 (BRCA2) variant associated PDAC, accounting for a percentage ranging from 4–10% in the various case series, actually have the ability to use Olaparib, a Poly ADP Ribose Polymerase (PARP) inhibitor, as 1st-line treatment maintenance after platinum-based induction chemotherapy [16]. Microsatellite instability-high (MSI-H) status represents an even smaller group of patients that could derive benefit from checkpoint inhibitors (namely pembrolizumab monotherapy) [17].

Treatment with different targeted therapies in an unstratified population of PDAC patients was associated with marginal improvement in survival, if any. For example, Moore et al. demonstrated a statistically significant improvement of survival using the association between gemcitabine plus erlotinib, a human epidermal growth factor receptor type 1 (HER1/EGFR) inhibitor, in unresectable, locally advanced, or metastatic pancreatic cancers that often overexpress HER1/EGFR [18]. On the other hand, Faloppi et al. underlined the prognostic and predictive negative role of lactate dehydrogenase (LDH) serum levels in advanced pancreatic cancer patients treated with sorafenib, a tyrosine kinase inhibitor [19].

However, the lack of proper histological specimens in the majority of patients makes molecular analysis of tumor tissues more difficult to perform. Particularly in patients without metastatic involvement, obtaining proper histologic tissue becomes a major issue, due to the anatomical location of the tumor and the pancreas itself. Indeed, a series of published papers have looked into other molecular determinants of PDAC progression, such as circulating tumor cells (CTCs), exosomes, and cell-free tumor deoxyribonucleic acid (DNA), to overcome this limitation [20,21,22].

Most of these studies have focused on molecular targets that have an already well-established role, with the assumption that the same molecular targets should have the same pathogenic role in PDAC as in other tumor types. This assumption has however proven wrong in most situations, prompting the need to clarify the role of established molecular markers specifically in PDAC patients.

Caveolin-1 (Cav-1) expression has been relatively recently described as an interesting molecular factor with a well-known mechanism of action: it is primarily involved in the formation of invaginations of the cell membrane that are crucial for vesicle formation and intra- and extra-cellular vesicle-mediated signaling. In particular, Boscher et al. showed the role of caveolin both as a promoter and inhibitor of different signaling pathways, assessing the impact of membrane domain localization on caveolin functionality in cell proliferation, survival, apoptosis, and migration [23].

There are a series of published papers that have associated the expression of Cav-1 to increased sensitivity to chemotherapy, and thus better prognosis. Most of these studies have been conducted in patients with breast and lung cancer, treatments which involve chemotherapy drugs that have to be internalized to be active. For breast cancer, nab-paclitaxel have been associated with highest intracellular concentration and activity whenever higher levels of Cav-1 expression were observed [24,25]. In particular, in metastatic breast cancer, Ricci et al. evidenced that higher tumor and lower stromal Cav-1 levels were significantly correlated with a longer PFS of nab-paclitaxel and gemcitabine [26]. Regarding lung cancer, in particular for advanced non-small cell lung cancer (NSCLC), Herrera et al. demonstrated the survival improvement using nab-paclitaxel in combination with carboplatin, especially in Cav-1 positive patients [27].

Even though this might have been proven to be true in breast and lung cancer, our results seem to suggest that in PDAC patients, high levels of expression of Cav-1 might have a completely opposite effect. Indeed, higher levels of Cav-1 seemed to be detected in metastases rather than in primary tumor histological samples, thus leading to the assumption that Cav-1 expression might change based on tumor growth pattern. Higher levels would be present in tumors that have a higher metastatic behavior. Albeit our population of patients received a heterogeneous number of first-line treatment options, patients treated with Gemcitabine+Nab-Paclitaxel did not have better survival compared with other treatments such as FOLFIRINOX, despite having been used in patients with high Cav-1 expression.

Campos et al. reported that Cav-1 might have different roles during pancreatic cancer progression, with a role as tumor suppressor factor in the earliest stages of PDAC development, but also as a promoter of enhanced aggressiveness in later stages of PDAC development [28]. Similar results have also been reported in prostate cancer, particularly in castration-resistant metastatic prostate cancer cells [29]. The overexpression of Cav-1 seems to be associated with low degree of differentiation, advanced clinical stage, and poor survival in prostate cancers [30]. High Cav-1 expression was also associated with a more aggressive behavior in melanoma cells [31,32] and significantly shorter survival in lung cancer patients. Particularly, Zhan et al. demonstrated that higher Cav-1 expression correlated with poorer lymph nodes stage and higher pathological TNM stage in lung adenocarcinoma (AC) patients, which was not found in lung squamous cell carcinoma (SCC) patients, impacting on prognosis [33].

Cav-1 expression would then be associated with a “double-edged” effect. In earlier stages of tumor development, it would act as a tumor suppressor factor, whereas in latter stages of tumor development it would help in enhancing tumor aggressiveness. For example, in breast cancer, stromal expression of Cav-1 has been associated with more aggressive behavior, impacting on metastatic spread and survival [34].

Indeed, looking at OS of patients enrolled in our analysis, higher levels of Cav-1 expression were associated with statistically significantly worse overall survival outcomes. This was maintained in spite of different regimens of treatment and patients’ clinical conditions at the beginning of first line treatment.

Based on these data, it would be interesting to assess whether Cav-1 expression, rather than just being used as a negative prognostic factor, could be used instead as a potential target for treatment. Incandronate for example, a bisphosphonate derivative, was found to be able to determine reduction of Cav-1 expression by inhibition of crucial steps in isoprenoid biosynthesis pathway leading to reduced production of geranylgeranylated-proteins [35]. Fenretinide, a synthetic derivative of retinoic acid, led to the down-regulation of caveolin-1 expression at the protein level in MG-63 and HOS osteosarcoma and A-172,LI,CRS-A2 glioblastoma cells, decreasing tumor aggressiveness and restoring chemosensitivity. [36].

Other Cav-1 inhibitors are currently used as drugs labeled for other indications, such as lovastatin, a common anti-cholesterol drug, and celecoxib, a rather commonly used not-steroidal anti-inflammatory drug. Guruswamy et al. reported that the anti-proliferative effect on colon cancer cell line HCT-116 determined by use of celecoxib and lovastatin was due to reduced Caveolin-1 expression in treated cells and the reduced activation of down-stream signaling pathways [37]. These promising preclinical data suggest that Cav-1 inhibitors may improve the efficacy of treatment of advanced PDAC and could be evaluated in clinical trials in association with chemotherapy.

Our study has several limitations. For example, tissue from both the primary tumor and metastatic site was not available in any patient, so we could not compare Cav-1 expression between primary tumor and metastases in the same patient. Moreover, tumor grading was available only for a few samples included in the study, so we could not assess the relationship between grading and Cav-1 expression.

## 5. Conclusions

Our study evidenced a negative prognostic role of Cav-1 in patients with metastatic/locally advanced unresectable PDAC, according to the literature. Moreover Cav-1 seems not to have any predictive value concerning response rates to different types of first line treatment. Therefore, further prospective research focused on the use of Cav-1 specific inhibitors should be conducted in order to increase the available therapeutic strategies and improve survival for advanced PDAC patients.

## Figures and Tables

**Table 1:** Characteristics of the patients.

Characteristic	N (%) Tot = 39
**Gender**	
Male	23 (59%)
Female	16 (41%)
Median age (range)	68 (31–86)
**ECOG**	
0	18 (46%)
1	14 (36%)
2	7 (18%)
**First Line Chemotherapy Regimen**	
Gemcitabine plus Abraxane	27 (69%)
FOLFIRINOX or FOLFOX	5 (12%)
Monotherapy	7 (17%)
**Stage at diagnosis**	
Locally advanced	17 (43%)
Metastatic	22 (67%)
**Metastatic sites**	
Liver	30 (76%)
Lung	8 (20%)
Peritoneum	4 (10%)
Bone	2 (5%)
Lymph nodes	13 (33%)

**Table 2:** Patients’ Outcomes.

Best Overall Response	N(%) Tot = 39
CR	0 (0%)
PR	8 (20%)
SD	10 (25%)
PD	17 (43%)
unknown	4 (10%)
median OS (months)	11.93
median PFS (months)	6.93

Complete Response (CR); Partial Response (PR); Stable Disease (SD), Progressive Disease (PD); Overall Survival (OS); Progression Free Survival (PFS).

**Table 3:** Sites of biopsy and tumoral Caveolin-1 expression.

Caveolin-1 Expression and Sampling Methods	N (%) Tot = 39
**Sites of biopsy**	
primary tumor	28 (70%)
liver metastasis	11 (30%)
**Sampling Method**	
Fine needle aspiration biopsy (FNAB) on primary tumor	12 (30%)
core biopsy on primary tumor	7 (18%)
core biopsy on liver metastasis	11 (28%)
surgical primary tumor sample	9 (24%)
**Tumoral Caveolin-1 expression (primary tumor)**	28
negative	13 (46%)
1+	2 (7%)
2+	10 (35%)
3+	2 (7%)
not evaluable	1 (4%)
**Tumoral Caveolin-1 expression (mestastatic sites)**	11
negative	0 (0%)
1+	0 (0%)
2+	6 (54%)
3+	5 (46%)

**Table 4:** Univariate analysis and multivariate analysis for Overall Survival.

Patients’ Characteristics		Univariate Analysis (OS)		mOS (Months)	Multivariate Analysis (OS)	
		HR (95% CI)	*p*		HR (95% CI)	*p*
Age	<75	1.46 (0.56–3.76)	0.43	12.72		NS
	≥75			7.15		
ECOG-PS	0		**0.0012**	16.02		NS
	1			9.87		
	2			2.46		
Chemotherapy regimen	Monotherapy	0.50 (0.15–1.67)	0.09	10.92		NS
	Combination			12.72		
Caveolin	2+/3+	5.35 (2.38–12.05)	**0.0001**	7.15	4.96 (2.02–12.16)	**0.0005**
	0/1+			22.3		

Values in bold letters show significant correlations.

**Table 5:** Univariate analysis and multivariate analysis for Progression Free Survival.

Patients’ Characteristics		Univariate Analysis (PFS)		mPFS (Months)	Multivariate Analysis (PFS)	
		HR (95% CI)	*p*		HR (95% CI)	*p*
Age	<75	1.5 (0.64–3.55)	0.34	6.23		NS
	≥75			4.75		
ECOG-PS	0		**0.0004**	6.72		NS
	1			7.87		
	2			2.10		
Chemotherapy regimen	Monotherapy	0.30 (0.07–1.33)	**<0.0001**	2.79	0.3 (0.10–0.9)	**0.03**
	Combination			7.25		
Caveolin	2+/3+	3.12 (1.44–6.72)	**0.0037**	4	2.81 (1.17–6.76)	**0.019**
	0/1+			8.59		

Values in bold letters show significant correlations.

## Data Availability

The data presented in this study are available on request from the corresponding author.
